# Lipid A Structural Divergence in *Rickettsia* Pathogens

**DOI:** 10.1128/mSphere.00184-21

**Published:** 2021-05-05

**Authors:** Mark L. Guillotte, Courtney E. Chandler, Victoria I. Verhoeve, Joseph J. Gillespie, Timothy P. Driscoll, M. Sayeedur Rahman, Robert K. Ernst, Abdu F. Azad

**Affiliations:** a Department of Microbiology and Immunology, School of Medicine, University of Maryland Baltimore, Maryland, USA; b Department of Microbial Pathogenesis, School of Dentistry, University of Maryland, Baltimore, Maryland, USA; c Department of Biology, West Virginia University, Morgantown, West Virginia, USA; UTMB

**Keywords:** *Rickettsia*, rickettsioses, spotted fever group, transitional group, typhus group, lipid A, lipopolysaccharide, pathogenesis

## Abstract

Species of *Rickettsia* (*Alphaproteobacteria*: *Rickettsiales*) are obligate intracellular parasites of a wide range of eukaryotes, with recognized arthropod-borne human pathogens belonging to the transitional group (TRG), typhus group (TG), and spotted fever group (SFG) rickettsiae. Growing in the host cytosol, rickettsiae pilfer numerous metabolites to make a typical Gram-negative bacterial cell envelope. The O-antigen of rickettsial lipopolysaccharide (LPS) is immunogenic and has been shown to tether the S-layer to the rickettsial surface; however, little is known about the structure and immunogenicity of the *Rickettsia* lipid A moiety. The structure of lipid A, the membrane anchor of LPS, affects the ability of this molecule to interact with components of the host innate immune system, specifically the MD-2/TLR4 receptor complex. To dissect the host responses that can occur during *Rickettsia in vitro* and *in vivo* infection, structural analysis of *Rickettsia* lipid A is needed. Lipid A was extracted from four *Rickettsia* species and structurally analyzed. *R. akari* (TRG), R. typhi (TG), and R. montanensis (SFG) produced a similar structure, whereas R. rickettsii (SFG) altered the length of a secondary acyl group. While all structures have longer acyl chains than known highly inflammatory hexa-acylated lipid A structures, the R. rickettsii modification should differentially alter interactions with the hydrophobic internal pocket in MD2. The significance of these characteristics toward inflammatory potential as well as membrane dynamics between arthropod and vertebrate cellular environments warrants further investigation. Our work adds lipid A to the secretome and O-antigen as variable factors possibly correlating with phenotypically diverse rickettsioses.

**IMPORTANCE** Spikes in rickettsioses occur as deforestation, urbanization, and homelessness increase human exposure to blood-feeding arthropods. Still, effective *Rickettsia* vaccines remain elusive. Recent studies have determined that *Rickettsia* lipopolysaccharide anchors the protective S-layer to the bacterial surface and elicits bactericidal antibodies. Furthermore, growing immunological evidence suggests vertebrate sensors (MD-2/TLR4 and noncanonical inflammasome) typically triggered by the lipid A portion of lipopolysaccharide are activated during *Rickettsia* infection. However, the immunopotency of *Rickettsia* lipid A is unknown due to poor appreciation for its structure. We determined lipid A structures for four distinct rickettsiae, revealing longer acyl chains relative to highly inflammatory bacterial lipid A. Surprisingly, lipid A of the Rocky Mountain spotted fever agent deviates in structure from other rickettsiae. Thus, lipid A divergence may contribute to variable disease phenotypes, sounding an alarm for determining its immunopotency and possible utility (i.e., as an adjuvant or anti-inflammatory) for development of more prudent rickettsiacidal therapies.

## Abstract (precis)

Spikes in rickettsioses occur as deforestation, urbanization, and homelessness increase human exposure to blood-feeding arthropods. Still, effective *Rickettsia* vaccines remain elusive.

## OBSERVATION

Lipopolysaccharide (LPS), an amphipathic molecule comprising the majority of the outer leaflet of the Gram-negative bacterial outer membrane, is composed of extracellular polysaccharide chains (O-antigen) linked to a membrane phosphoglycolipid (lipid A) by a core oligosaccharide. Depending on its structure, lipid A can be a potent activator of the mammalian immune system through detection by MD-2/TLR4 (1, 2) and the noncanonical inflammasome (3, 4). However, not all lipid A structures are equal in their ability to activate these mammalian cellular receptors (5); for instance, some bacterial pathogens employ lipid A modification as a mechanism of immune evasion when infecting a mammalian host (6, 7). Given the importance of lipid A for membrane integrity, resistance to antibiotics, and use as a vaccine adjuvant, it is crucial to understand the structure and immunostimulatory potential of lipid A from Gram-negative pathogens.

**FIG 1:**
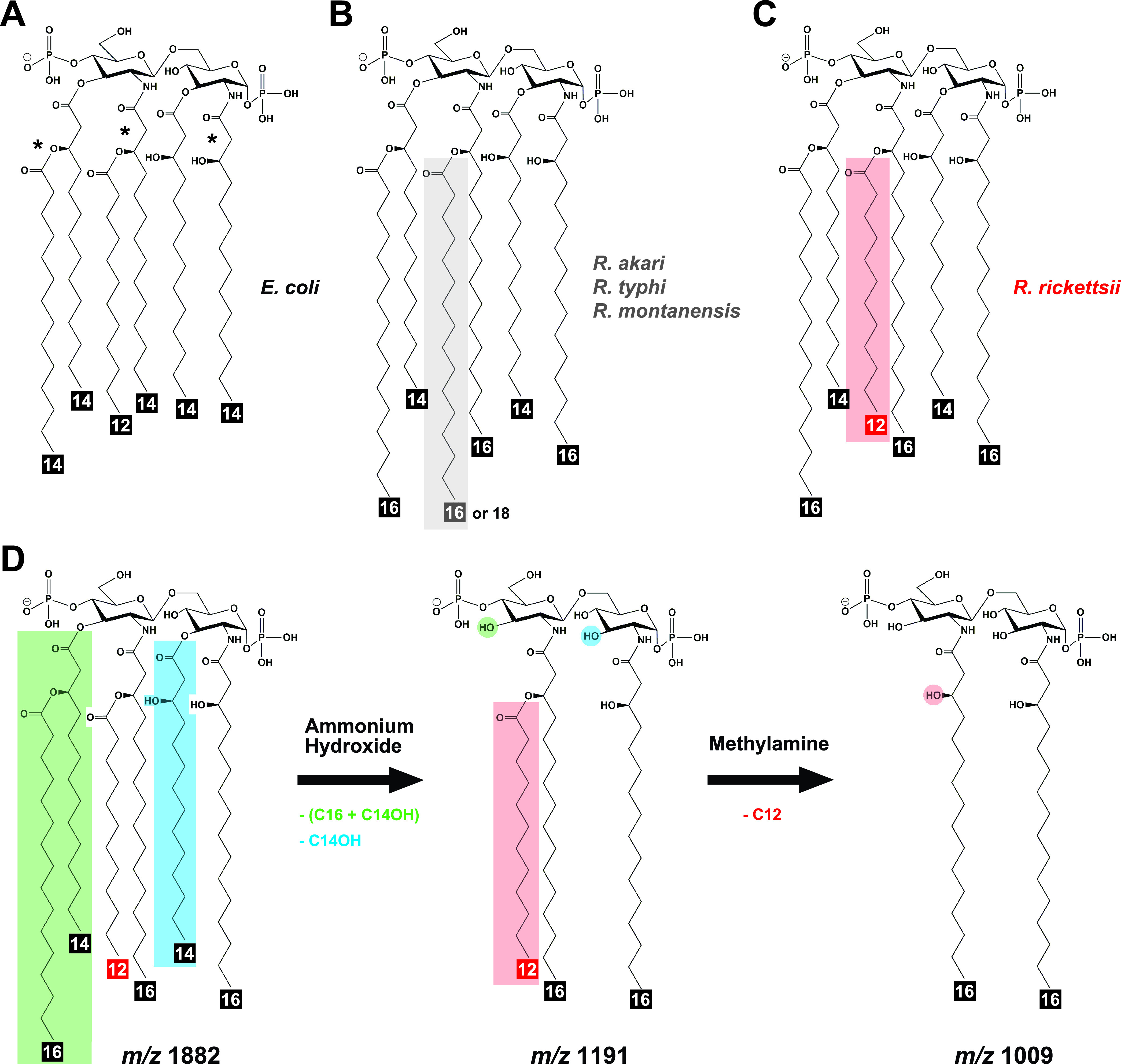
Variable acyl chain lengths in *Rickettsia* lipid A. (A) The structure of the highly inflammatory lipid A of E. coli. Asterisks depict acyl chains that diverge in length in *Rickettsia* lipid A. (B) Structure of lipid A isolated from *R*. *akari* strain Hartford, R. typhi strain Wilmington, and R. montanensis strain M5/6 during Vero 76 cell infection. (C) Structure of lipid A isolated from R. rickettsii strains Sheila Smith and Iowa during Vero 76 cell infection (for full spectra, see Fig. S1 in the supplemental material). (D) Schematic representation of analytical methods used to determine fatty acid compositions, with the R. rickettsii lipid A shown as an example. Lipid A from R. rickettsii strain Sheila Smith was subjected to sequential release of fatty acids and analyzed at each step with MALDI-TOF analysis (for full spectra, see Fig. S2). *m/z*, mass-to-charge ratio of lipid A ions identified during MALDI-TOF analysis.

Species of *Rickettsia*, Gram-negative obligate intracellular *Alphaproteobacteria*, are metabolic parasites of a wide range of eukaryotic hosts (8). Across the *Rickettsia* tree, agents of human disease from the transitional group (TRG), typhus group (TG), and spotted fever group (SFG) rickettsiae are interspersed with numerous invertebrate and protist endosymbionts, most with unknown pathogenicity. All described rickettsioses are vector-borne diseases that differ in their severity of illness and clinical manifestations (9), facts undoubtedly linked to variability in the *Rickettsia* secretome (10) and O-antigen epitopes (11, 12). *Rickettsia* O-antigen contains the sugar quinovosamine (11–13), and transposon-mediated disruption of an epimerase involved in its production abrogates S-layer formation, dampens pathogenicity, and abolishes recognition by bactericidal antibodies (14). While the immunopotency of the lipid A moiety of *Rickettsia* LPS remains unknown, it could be proinflammatory given MD-2/TLR4 and noncanonical inflammasome activation during infection (15–20). However, the lone published *Rickettsia* lipid A structure (an unreported strain of Rickettsia typhi, the agent of murine typhus) revealed a bisphosphorylated hexa-acylated structure with acyl chains ranging from C14 to C18 in length (21), much longer than the C12-C14 chains of the highly inflammatory hexa-acylated lipid A of Escherichia coli (Fig. 1A). These collective observations warrant determining *Rickettsia* lipid A structures for phenotypically diverse species to assess if any structural variability correlates with disease severity.

10.1128/mSphere.00184-21.1 FIG S1 MALDI-TOF analysis of *Rickettsia* lipid A. Lipid A microextraction from rickettsial cultures was performed as previously described (A. El Hamidi, A. Tirsoaga, A. Novikov, A. Hussein, et al., J Lipid Res 46:1773–1778, 2005; A. J. Scott, B. Flinders, J. Cappell, T. Liang, et al., Pathog Dis 74, 2016; A. Tirsoaga, A. El Hamidi, M. B. Perry, M. Caroff, et al., J Lipid Res 48:2419–2427, 2007). See the text for additional details. (A) R. typhi strain Wilmington, (B) R. montanensis strain M5/6, (C) *R*. *akari* strain Hartford, and (D) R. rickettsii strain Sheila Smith. Insets identify the vector host and relative virulence to humans. Peak labels are masses of singly charged ions. Major peaks are indicated by colored labels (A to C, gray; D, red), with minor peaks (likely representing heterogeneity in fatty acid incorporation) noted by dashed lines. Download FIG S1, PDF file, 1.3 MB. Copyright © 2021 Guillotte et al. 2021 Guillotte et al. https://creativecommons.org/licenses/by/4.0/ This content is distributed under the terms of the Creative Commons Attribution 4.0 International license.

10.1128/mSphere.00184-21.2 FIG S2 Fatty acid analysis of *Rickettsia* lipid A. (A, D, G, J, and M) Lipid A of *R. akari* strain Hartford, R. typhi strain Wilmington, R. montanensis strain M5/6, R. rickettsii strain Sheila Smith, and R. rickettsii strain Iowa was subjected to sequential fatty acid release prior to MALDI-TOF analysis of daughter molecules (A. El Hamidi, A. Tirsoaga, A. Novikov, A. Hussein, et al., J Lipid Res 46:1773–1778, 2005; A. Tirsoaga, A. El Hamidi, M. B. Perry, M. Caroff, et al., J Lipid Res 48:2419–2427, 2007). (B, E, H, K, and N) Primary ester-linked fatty acids (3′ position, green; 3 position, blue), which are more readily released than secondary ester-linked fatty acids, were liberated using mild alkali treatment with ammonium hydroxide, yielding predominate ions of either *m/z* ∼1,247/1,273 (gray for palmitate/stearate in *R. akari*, R. typhi, and R. montanensis) or *m/z* 1,191 (red for laurate in R. rickettsii) that are tri-acyl daughter molecules. (C, F, I, L, and O) The remaining secondary ester-linked fatty acid attached to the hydroxypalmitate at the 2′ position was liberated using a second alkaline reaction with methylamine, yielding the di-acyl daughter ion at *m/z* 1009 for all analyzed strains. Structures (insets) depict the major lipid A ion in each spectrum, with minor peaks (dashed lines) likely representing heterogeneity in fatty acid incorporation. Download FIG S2, PDF file, 2.5 MB. Copyright © 2021 Guillotte et al. 2021 Guillotte et al. https://creativecommons.org/licenses/by/4.0/ This content is distributed under the terms of the Creative Commons Attribution 4.0 International license.

To investigate possible lipid A diversity between rickettsiae, we isolated and analyzed lipid A from three human pathogens (*R. akari*, R. typhi, and R. rickettsii) and a nonpathogen (R. montanensis), all grown in Vero cell cultures. Lipid A extracted directly from infected-Vero cells for *R. akari*, R. typhi, and R. montanensis produced major ions at *m/z* 1,936 that are largely consistent with the published R. typhi lipid A structure (21) and likely represent bisphosphorylated hexa-acyl structures (Fig. 1B). However, there is considerable variance between species in the number and intensity of minor lipid A ions (*m/z* 1,963, 1,909, and 1,882) that likely represents heterogeneity in fatty acid (FA) incorporation (see Fig. S1 in the supplemental material) known to occur in some bacteria (22). In contrast, the Rocky Mountain spotted fever agent, R. rickettsii, produces a lower-molecular-weight lipid A molecule (major ion, *m/z* 1,882) (Fig. 1C), corresponding to a loss of four carbons from one or more FA chains (Fig. 1D). Sequential FA release by alkaline treatment indicates a laurate (C12) on 2′-hydroxypalmitate (C16-OH) as opposed to palmitate/stearate (C16/C18) in the other *Rickettsia* structures (Fig. S2).

**FIG 2:**
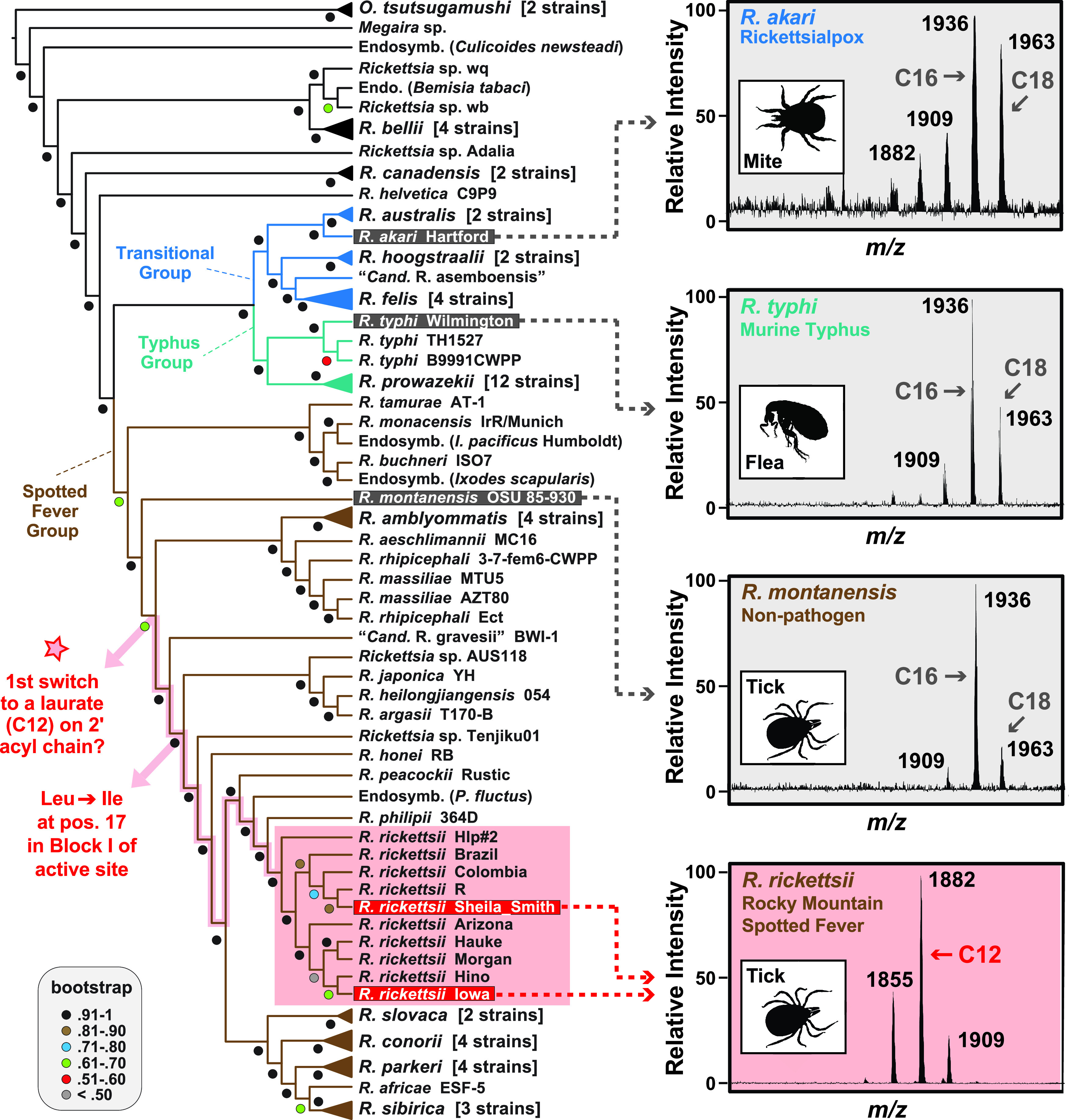
Evolution of structural variability in *Rickettsia* lipid A. Genome-based phylogeny on the left was estimated as previously described (25). Mass spectra on the right depict MALDI-TOF analyses of lipid A extracted from *R*. *akari* strain Hartford, R. typhi strain Wilmington, R. montanensis strain M5/6, and R. rickettsii strains Sheila Smith and Iowa (for full spectra, see Fig. S1 in the supplemental material). Peak labels are masses of singly charged ions; arrows denote major peaks. Insets show typical arthropod vectors. The star on the phylogeny indicates the earliest point in SFG rickettsia evolution where a switch from palmitate/stearate (C16/18) to laurate (C12) on lipid A 2′ hydroxypalmitate could have occurred. The node reflecting a switch to a conserved Ile in position 17 of block I of the LpxL active site is also noted (see Fig. S4 for more details).

The underlying mechanisms for acyl chain heterogeneity within species and divergence between R. rickettsii and other rickettsiae are not readily apparent. The enzymes for lipid A biosynthesis are highly conserved across rickettsiae (Fig. S3), including the motifs that function as a hydrocarbon ruler in late acyltransferase LpxJ (23). However, inspection of analogous motifs in late acyltransferase LpxL revealed an active-site substitution (Leu-Ile) conserved in SFG rickettsiae that diverge after *R*. *gravesii* (Fig. 2, Fig. S4). The potential for this modified LpxL active site to incorporate shorter 2′ acyl chains into *Rickettsia* lipid A awaits experimentation. Despite conserved FA synthesis machinery, malonyl-ACP is synthesized in rickettsiae using host-derived precursors and cofactors (24), making FA availability and incorporation contingent on host metabolic burden during infection. Variance in FA acyl chain length may also reflect engrained flexibility for adapting to divergent cellular environments encountered across invertebrate and vertebrate hosts, making it a priority to assess lipid A structures directly from arthropod and mammalian infections.

10.1128/mSphere.00184-21.3 FIG S3 Raetz pathway for biosynthesis of lipid A in rickettsiae. Rickettsiae lack enzymes for amino sugar metabolism and synthesis of pentose phosphates, necessitating the acquisition of host *N*-acetylglucosamine-1-P and ribose-5-P to initiate biosynthesis of lipid IV(A) and 3-deoxy-d-manno-octulosonate (Kdo), respectively (T. P. Driscoll, V. I. Verhoeve, M. L. Guillotte, S. S. Lehman, et al., mBio 8:e00859-17, 2017). Using these two pilfered metabolites, 14 enzymes (conserved in *Rickettsia* genomes, inset at top) and three cofactors (ATP, UTP, and phosphoenolpyruvate) are required for Kdo_2_-lipid A biosynthesis. Kdo_2_ is not shown on the final structure, as our lipid A microextraction protocol removed Kdo residues prior to lipid A analysis (the structure of Kdo_2_ and the inner and outer core oligosaccharide have not been determined for *Rickettsiae* to date). Enzymes contain locus tags for R. typhi strain Wilmington enzymes that were used in blastp searches against the NCBI *Rickettsia* database (taxid 780) to confirm the strict conservation of all 14 CDS in the five strains analyzed in this study. Searches were performed with composition-based statistics, with no filter used. Default matrix parameters (BLOSUM62) and gap costs (existence, 11; extension, 1) were implemented, with an inclusion threshold of 0.005. Download FIG S3, PDF file, 1.4 MB. Copyright © 2021 Guillotte et al. 2021 Guillotte et al. https://creativecommons.org/licenses/by/4.0/ This content is distributed under the terms of the Creative Commons Attribution 4.0 International license.

10.1128/mSphere.00184-21.4 FIG S4 Comparative analysis of *Rickettsia* LpxL enzymes. Lipid A biosynthesis in E. coli utilizes the late acyltransferase LpxL to add a laurate (C12) to the 2′ hydroxymyristate (D. A. Six, S. M. Carty, Z. Guan, and C. R. H. Raetz, Biochemistry 47:8623–8637, 2008). Our work on *Rickettsia* lipid A indicates that LpxL enzymes add either a palmitate or stearate on the 2′-hydroxypalmitate (*R. akari*, R. typhi, and R. montanensis) or a laurate (C12) on the 2′-hydroxypalmitate (R. rickettsii strains Sheila Smith and Iowa). (A) Multiple-sequence alignment of LpxL homologs using MUSCLE (default parameters) (R. C. Edgar, Nucleic Acids Res 32:1792–1797, 2004) indicates strong conservation across the entirety of the proteins (the proteins from R. rickettsii strains Sheila Smith and Iowa are identical). Amino acid similarity (% identity) between homologs is shown below the alignment. Structural modeling of the R. typhi LpxL protein to Acinetobacter baumannii LpxM (PDB entry 5KNK) using Phyre2 (L. A. Kelley and M. J. E. Sternberg, Nat Protoc 4:363–371, 2009) indicates strong conservation within the three blocks recognized within GPAT, LPAAT, DHAPAT, and LPEAT acyltransferases (J. Yao and C. O. Rock, Biochim Biophys Acta 1831:495–502, 2013; T. M. Lewin, P. Wang, and R. A. Colema, Biochemistry 38:5764–5771, 1999). Active-site residues within blocks are colored according to charge. Asp193 is proposed to participate in the active-site charge relay system with His122 (R. J. Heath, C. O. Rock, J Bacteriol 180:1425–30, 1998; A. F. Neuwald, Curr Biol 7:R465-R466, 1997). There is strict conservation within the regions analogous to the deep hydrophobic cleft of LpxM predicted to function as a hydrocarbon ruler (D. Dovala, C. M. Rath, Q. Hu, W. S. Sawyer, et al., Proc Natl Acad Sci U S A 113:E6064–E6071, 2016). (B) Mapping of character states for the five unique R. rickettsii LpxL residues over the *Rickettsia* phylogeny from Fig. 1. Shared residues between R. rickettsii strains and other rickettsiae are highlighted yellow. The strict conservation of Ile130 within block I of the LpxL active site is illustrated at left. All other information follows the description for Fig. 1. Download FIG S4, PDF file, 0.7 MB. Copyright © 2021 Guillotte et al. 2021 Guillotte et al. https://creativecommons.org/licenses/by/4.0/ This content is distributed under the terms of the Creative Commons Attribution 4.0 International license.

The membrane or immunological function of shorter 2′ secondary acyl chains in derived SFG rickettsiae remains to be determined. If other human pathogens in this lineage diverging after R. montanensis (Fig. 2, red star) have acyl chain lengths similar to those of R. rickettsii, this lipid A structure may serve as a candidate drug target or vaccine adjuvant for treatment of these particular SFG rickettsioses. Furthermore, determining lipid A structures for other species throughout the rickettsial tree stands to illuminate additional structural diversity that bolsters joining lipid A with the secretome and O-antigen as variable factors defining phenotypically diverse rickettsioses.

Rickettsiae are rare among obligate intracellular bacteria in that they lyse the phagosome and colonize the host cytosol, risking inordinate metabolic thievery in the face of host surveillance systems (24). The pilfering of host metabolites for cell envelope synthesis entangles rickettsial growth and virulence. Further characterization of rickettsial lipid A, particularly its immunopotency, is imperative for advancing knowledge on host-pathogen interactions and a highly unique mode of obligate intracellular parasitism.

### Bacterial strains and cell culture.

Vero 76 cells (African green monkey kidney; ATCC CRL-1587) were maintained in Dulbecco’s modification of Eagle’s medium (DMEM with 4.5 g/liter glucose and 480 l-glutamine) supplemented with 10% heat-inactivated fetal bovine serum (FBS) at 37°C with 5% CO_2_. Rickettsiae were propagated in Vero 76 cells grown in DMEM (supplemented with 5% FBS at 34°C with 5% CO_2_) for 48 to 72 h until confluence before harvesting. Rickettsial cultures were partially purified by mild sonication (one 10-s pulse; power output 6) followed by 5.0-μm filtration and collected by gentle centrifugation (2,000 × *g*; 15 min). Pellets were washed once in ultrapure water prior to lipid A extraction.

### Lipid A extraction.

Lipid A microextraction from rickettsial cultures initiated by placing pellets from 1 to 4 confluent T75 cm^2^ culture flasks (maintained as described above) in 400 μl of solution (5 parts isobutyric acid, 3 parts 1 M NH_4_OH) and heated at 100°C for 1 h followed by a 15-min incubation on ice and centrifugation at 2,000 × *g* for 15 min. The lipid A-containing bottom layer of supernatant was collected, mixed in equal parts with H_2_O, frozen, and then lyophilized. Contaminants were washed from the dried material by two rounds of methanol (MeOH) washes (1 ml MeOH with sonicating and pelleting at 10,000 × *g* for 5 min). The final product was reconstituted in 50 μl chloroform-MeOH-H_2_O (2:1:0.25) along with 4 to 8 grains of Dowex ion exchange resin (ThermoFisher), incubated at room temperature with vortexing (5 min). Solubilized lipid A (1 to 2 μl) was spotted onto a stainless steel target plate with 1 μl of Norharmane matrix (10 mg/ml in 2:1 chloroform:methanol) for matrix-assisted laser desorption ionization (MALDI) analysis on a Bruker microflex MALDI-time of flight mass spectrometry (TOF MS) instrument in negative-ion mode calibrated with Agilent tuning mix (Santa Clara, CA), and data were processed in flexAnalysis (Bruker Daltonics). All microextraction chemicals were obtained from Sigma-Aldrich (St. Louis, MO).

### Sequential fatty acid release by alkaline treatment.

To liberate primary ester-linked fatty acids, lipid A samples were suspended in 100 μl of 28% NH_4_OH and incubated at 50°C for 5 h with occasional vortexing. To liberate secondary fatty acids, lipid A or treated derivatives were suspended in 40% methylamine and incubated at 50°C for 3 h. Ultrapure H_2_O (100 μl) was added before samples were frozen and lyophilized. Final products were reconstituted in chloroform-MeOH-H_2_O (2:1:0.25) and analyzed by MALDI-TOF. The entire workflow described above was performed in triplicate for all five strains.
