# Supplementary figures and images for: Lipid A Structural Divergence in Rickettsia Pathogens
Source: mSphere. 2021 May 5;6(3):e00184-21. doi: 10.1128/mSphere.00184-21 (PMC8103985; doi:10.1128/mSphere.00184-21)

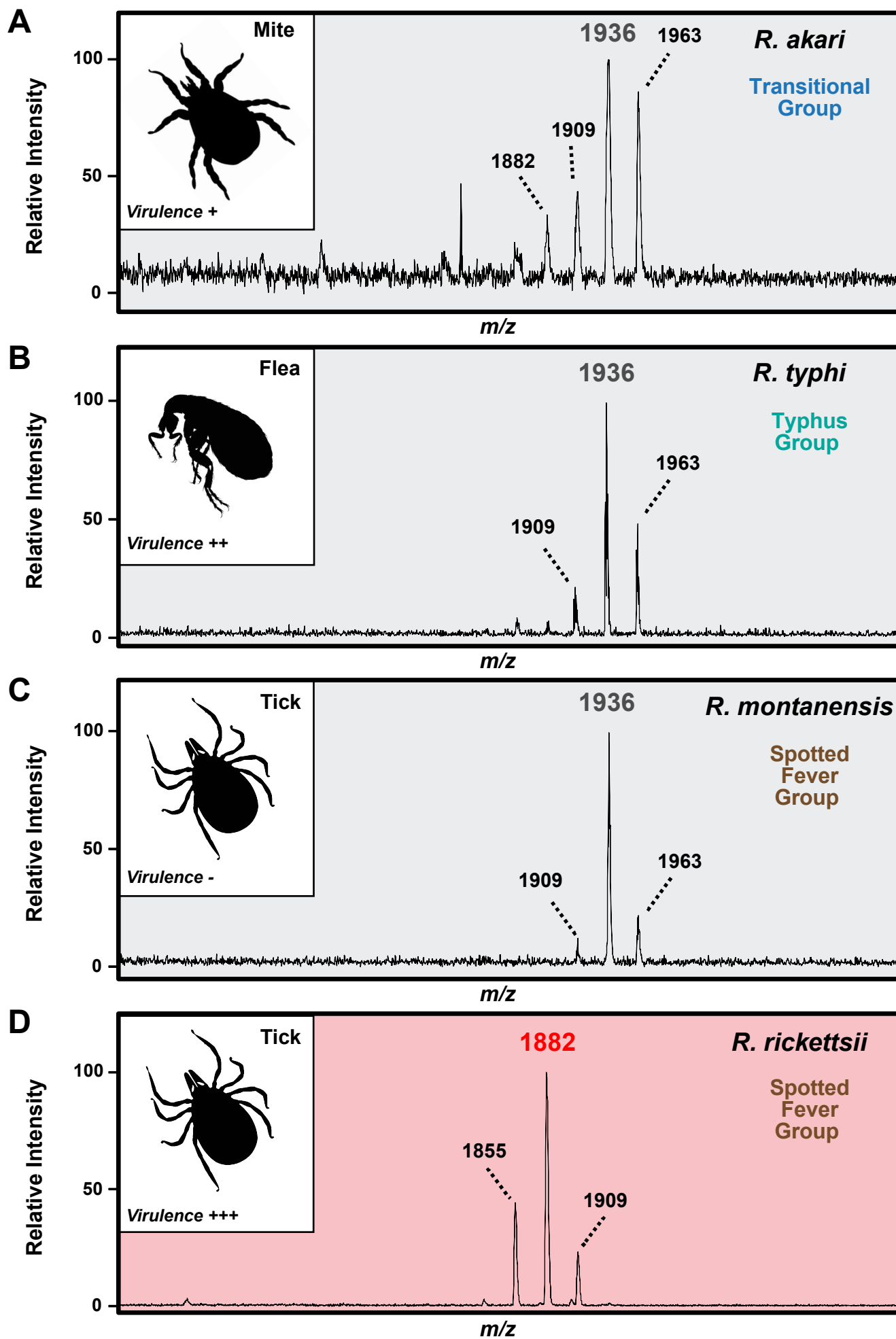

Supplement: FIG S1 [file mSphere.00184-21-sf001.pdf]

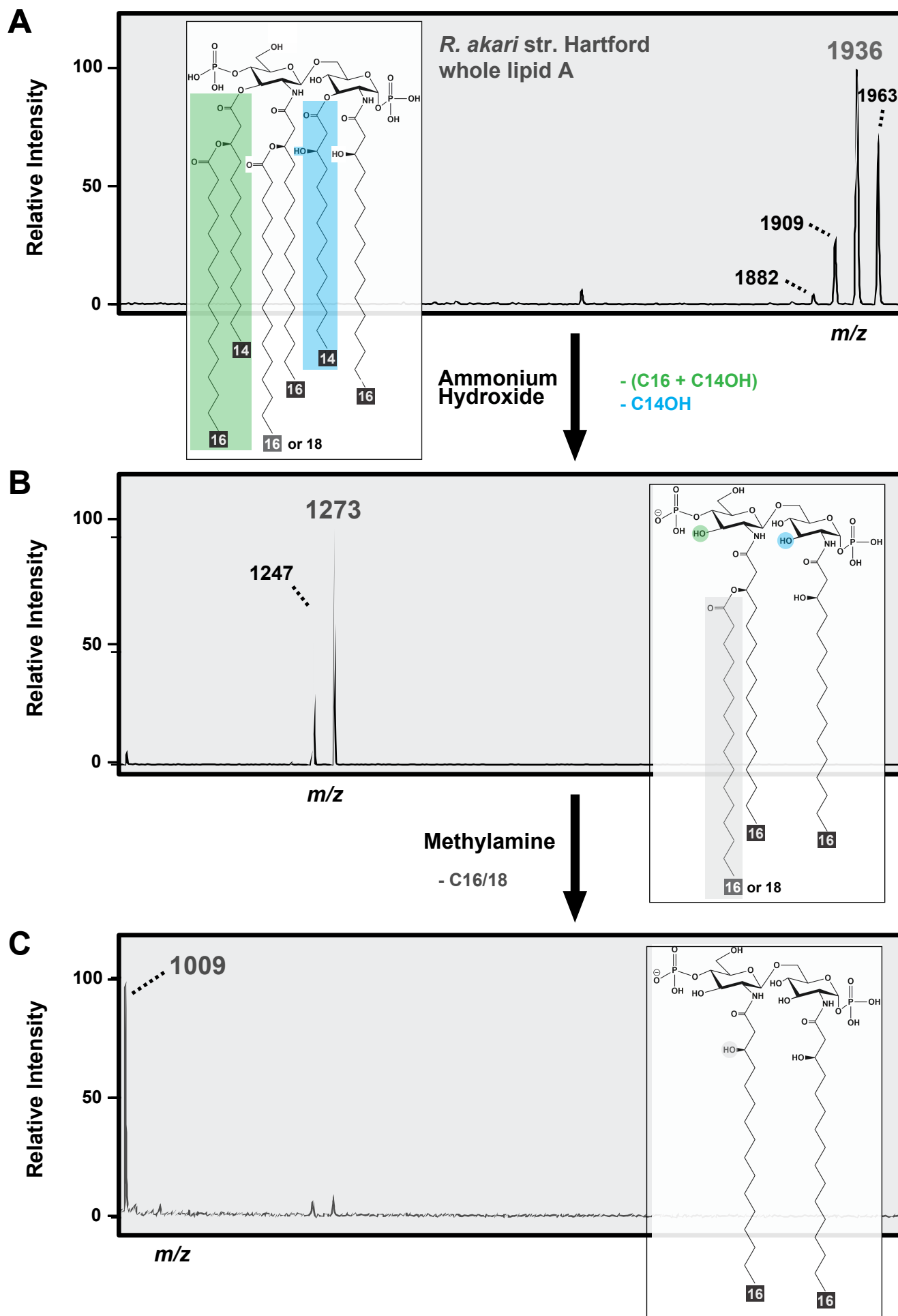

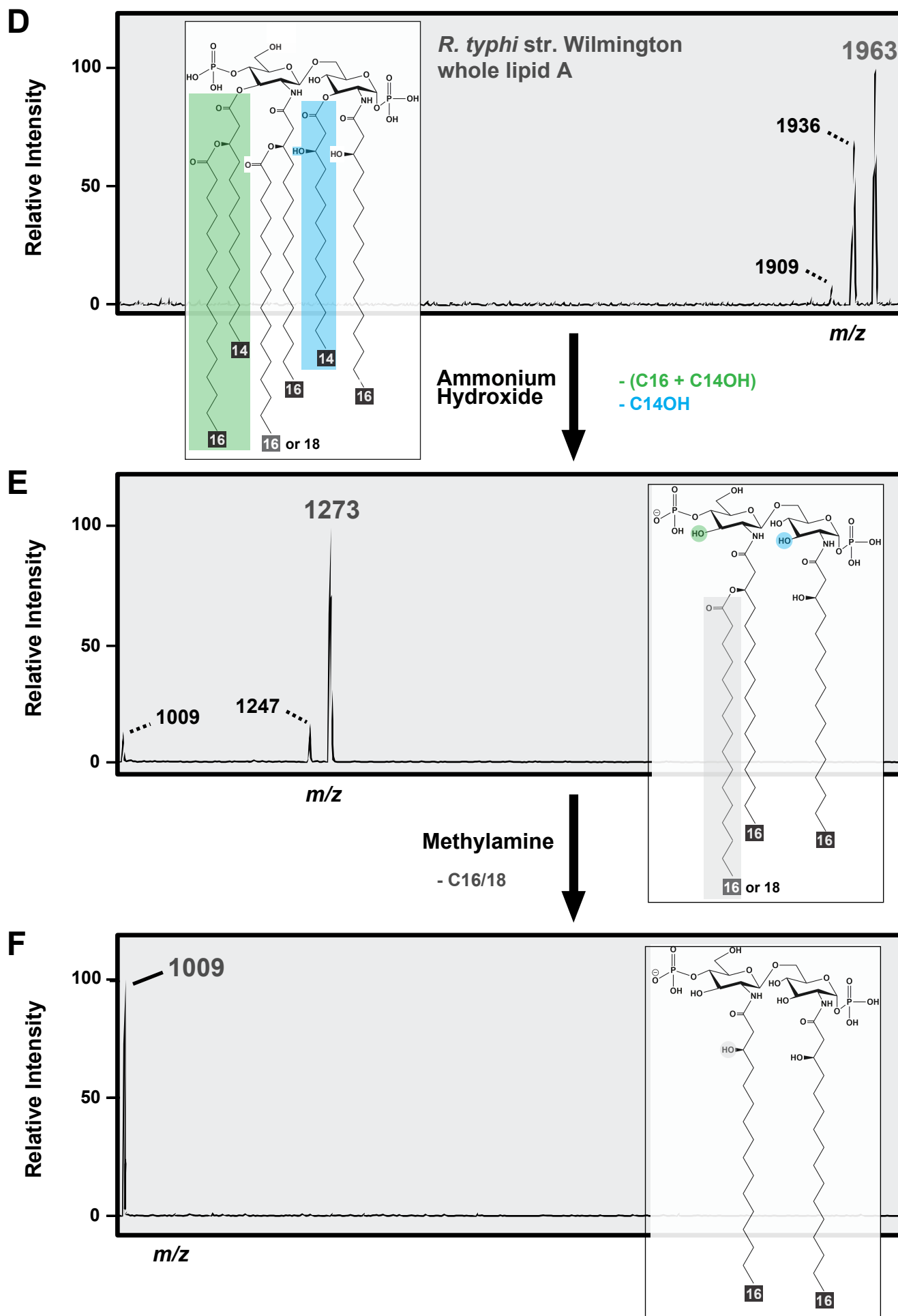

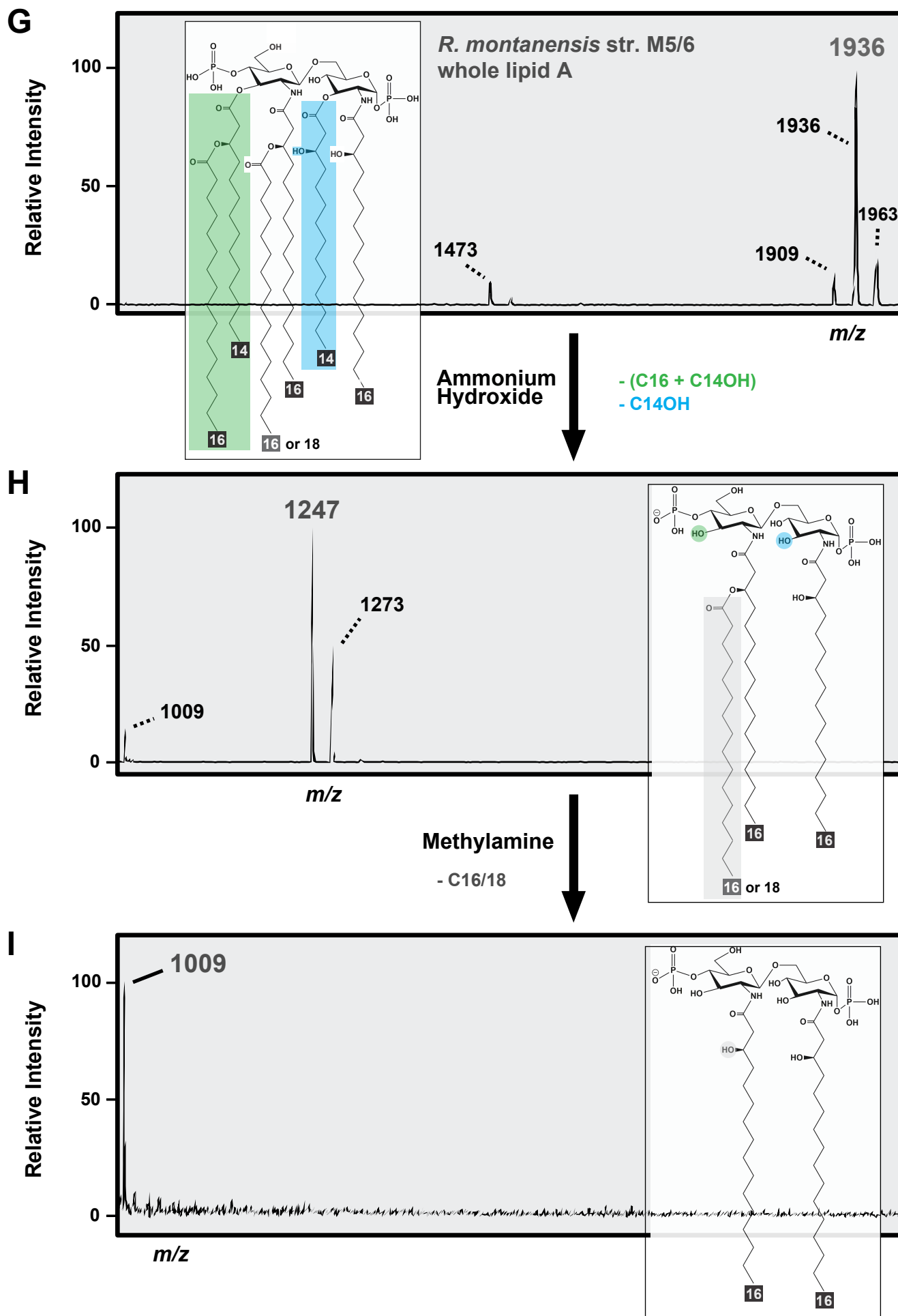

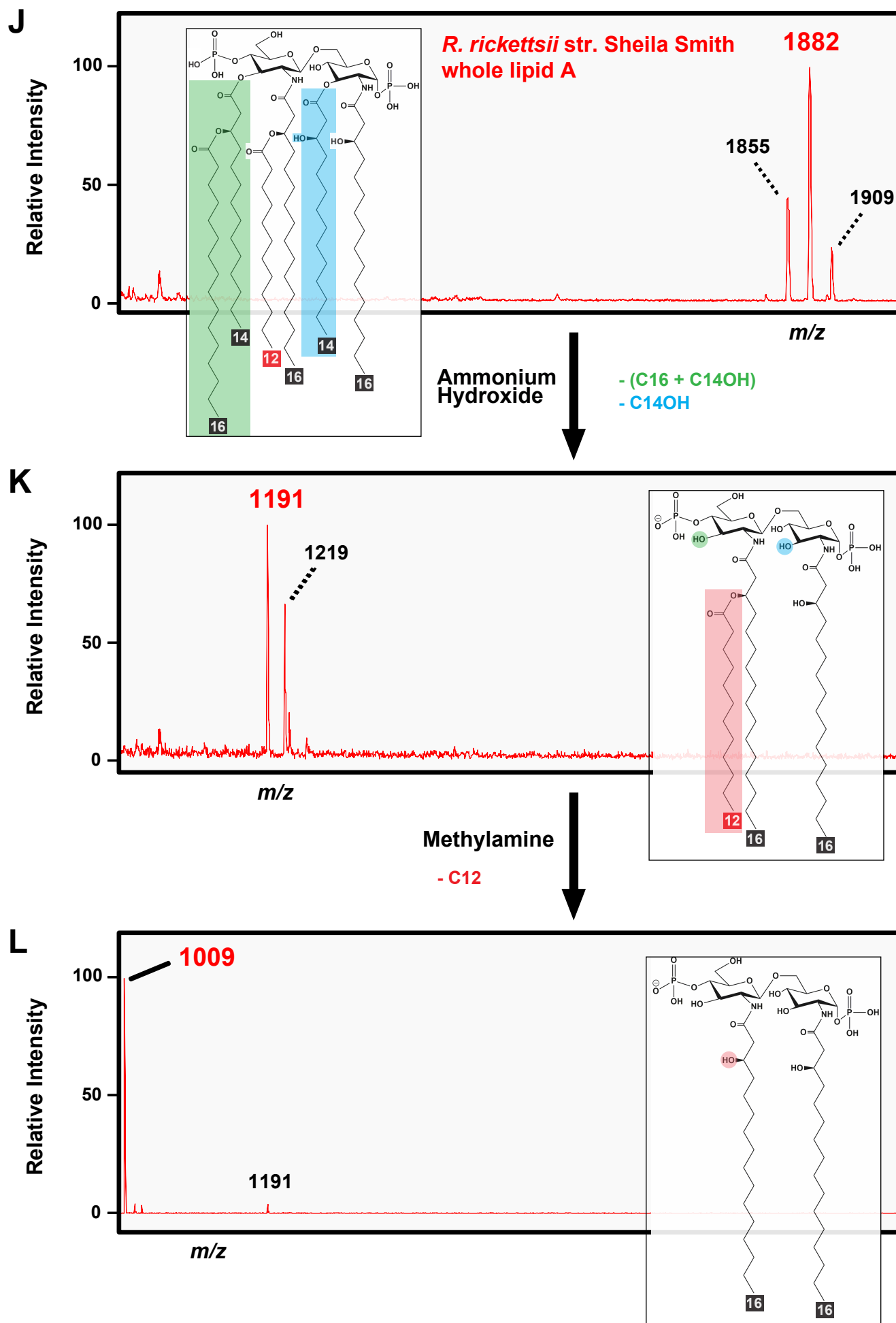

M

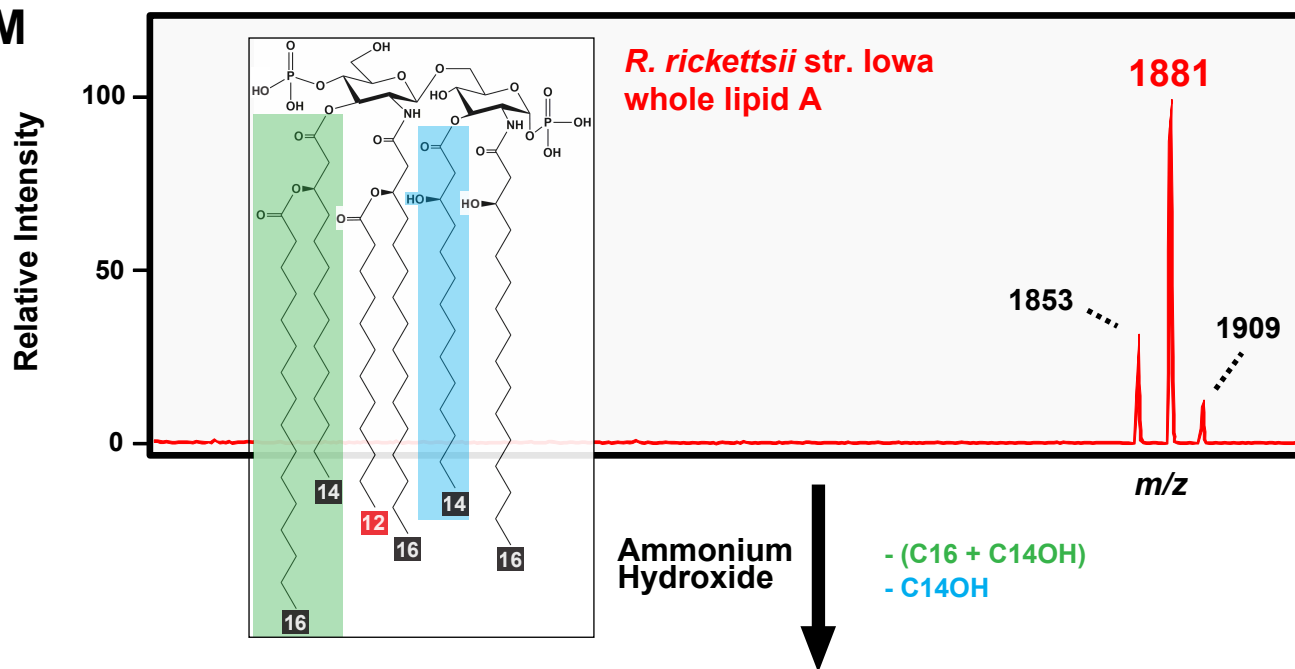

N

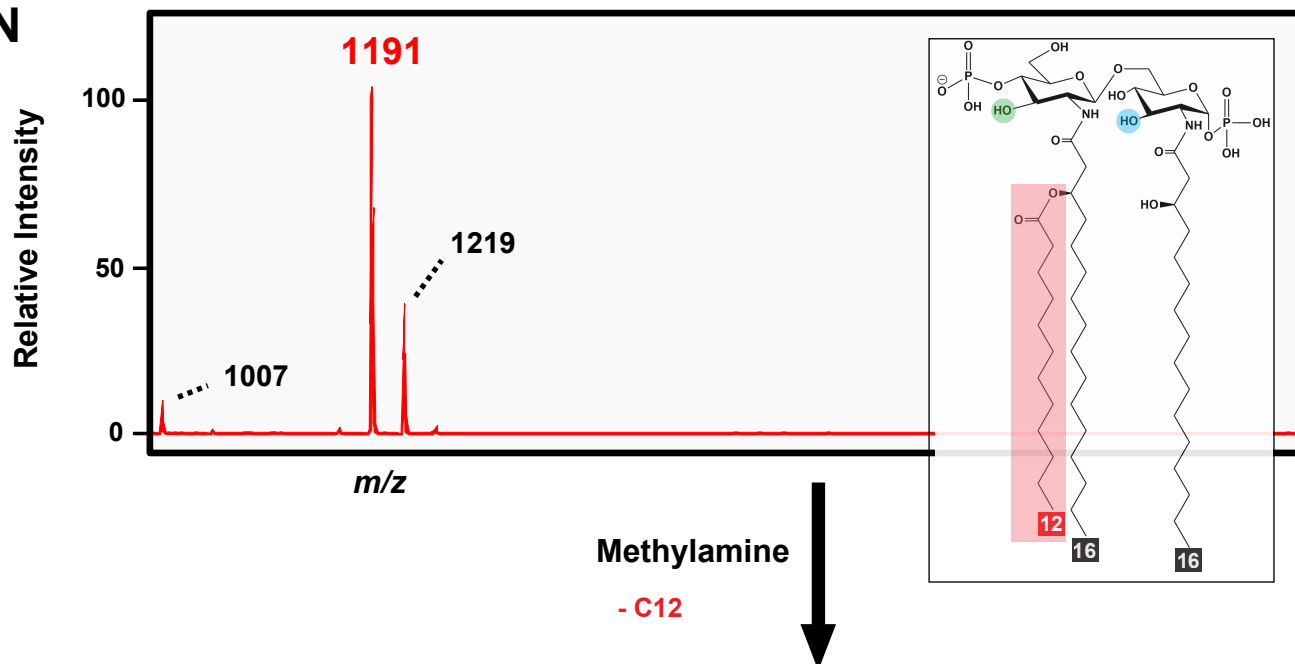

O

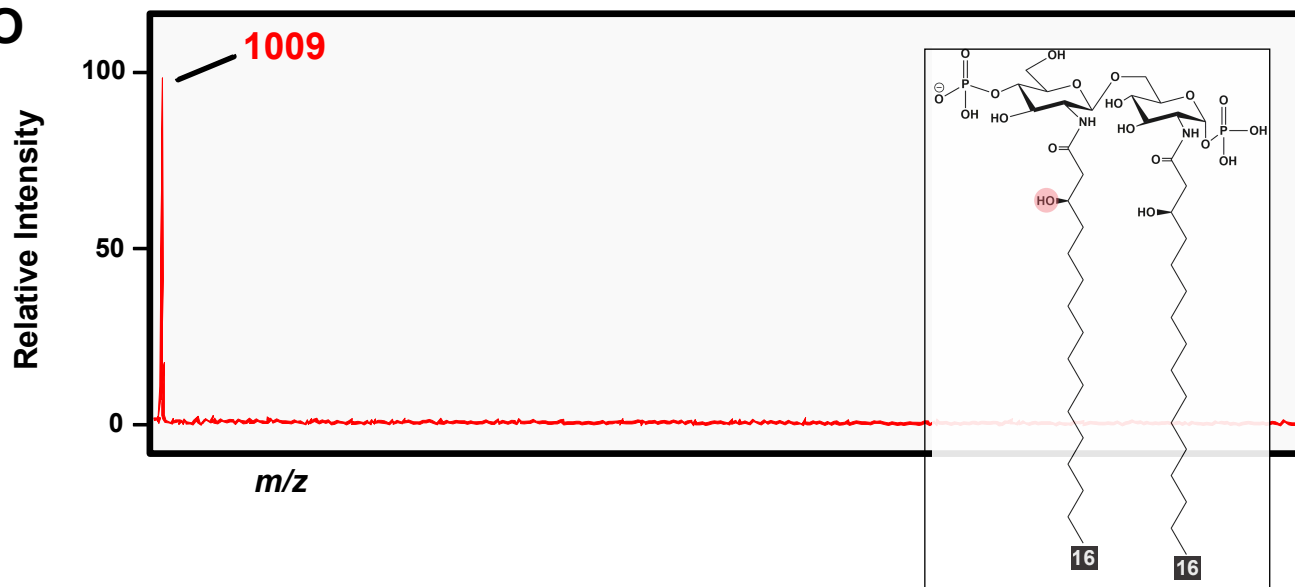

Supplement: FIG S2 [file mSphere.00184-21-sf002.pdf]
