# Supplementary material for: Lipid A Structural Divergence in Rickettsia Pathogens
Source: mSphere. 2021 May 5;6(3):e00184-21. doi: 10.1128/mSphere.00184-21 (PMC8103985; doi:10.1128/mSphere.00184-21)

FIG. S4

A

```
                                                     Block I
R. akari        MKKFLKKLRYLIEYFIVVIFLKVIGIFGVDKAADICSFIARKVGILFAVNKIAKRNIKAV   60
R. typhi        MKKFLKKLRYLIEYFIVIIFLKVIGIFGIDKASDICSFIARKVGILFAVNKIAKRNIKAV   60
R. montanensis  MKKFLKKLRYLIEYFIVVIFLKVIGIFGVDKAADICSFIARKVGILFAVNKIARRNIKVV   60
R. rickettsii   MKKFLKKLRYLIEYFIVVIFLKVIGIFGVDKAADICSFIARKVGILFAVNKIARRNIKVV   60
                *****************:**********:***:********************.****.*

R. akari        FGDMCDVEKIIDKTWDNFGRFIGESAYVNKMSDAELEHRAEIIGIENIRKLGERPFLLFS   120
R. typhi        FGDMYDVEKIIDQTWDNFGRFIGEFTYVDKMDESELERRIEIIGKENIQKLEDQPFLLFS   120
R. montanensis  FGDMCDVEKIIDQTWDNFGRFIGEFTYVGKMDEVELERRIEIIGIENIKKLEGQPFLLFS   120
R. rickettsii   FGYMCDVEKIIDQTWDNFGRFIGEFTYVSKMDEAELERRIAIIGIENIKKLEGQPFLLFS   120
                ** * *******:*********** :**.**.: ***.*  *** ***.**  .******

                  Block I                                Block II
R. akari        GHFANWDIGLHLLHKSYSKFAVIYRKANNPYVNKLINESRAGDKLRLIPKGPEGRRALVR   180
R. typhi        GHFANWDISLKVLKKYYPKVAVIYRKANNPYVNKLVNESRAGDKLRLIPKGSEGIRALVS   180
R. montanensis  GHFANWDISLKLLHKFYPKVAVIYRKANNPYVNKLVNESRAGDKLRLIPKGPEGSRALVR   180
R. rickettsii   GHFANWDISIKILHKFYPKVAVIYRKANNPYVNKLVNESRAGDKLRLIPKGPEGSRALVR   180
                ********.:::*:* *.*.***************:***************.** ****

                     Block III
R. akari        AIKDGESIVMLVDQKMNDGIEVPFLGHPAMTANAIAKIALQYKYTIIPCQIIRTKGSYFK   240
R. typhi        AIKEGESIVMLVDQKMNDGIEVPFLGRPSMTASAIAKIALQYKYPIIPCQIIRTKGSYFK   240
R. montanensis  AIKESEPIVMLVDQKMNDGIEVPFLGHPAMTASAIAKIALQYKYPIIPCQIIRTKGSYFK   240
R. rickettsii   AIKESEAIVMLVDQKMNDGIEVPFLGHPAMTASAIAKIALQYKYPIIPCQIIRTKGSYFK   240
                ***:.*.*******************.*:***.***********.***************

R. akari        VIVHPQLEFEQTGDNKADCYNIMLNINQMLGEWVKQNPSQWFWFHNRWKK             290
R. typhi        VIVHPQLKFEQTGDNKVDCYNIMFYINQILGEWVKQNPAQWFWFHNRWKK             290
R. montanensis  VIVHPQLKFEQTGDNKADCYNIMLNINQMLGEWVKQNPSQWFWFHNRWKK             290
R. rickettsii   VIVHPQLKLEQTGDNKADCYNIMLNINQMLGEWVKQNPAQWFWFHNRWKQ             290
                *******::*******.******: ***:*********:**********:
```

R. akari	-----	86.21	90.34	88.28
R. typhi	86.21	-----	91.03	89.66
R. montanensis	90.34	91.03	-----	96.55
R. rickettsii	88.28	89.66	96.55	-----

Active site pos. (blue) or neg. (red)

(yellow) Residue unique to *R. rickettsii*

B

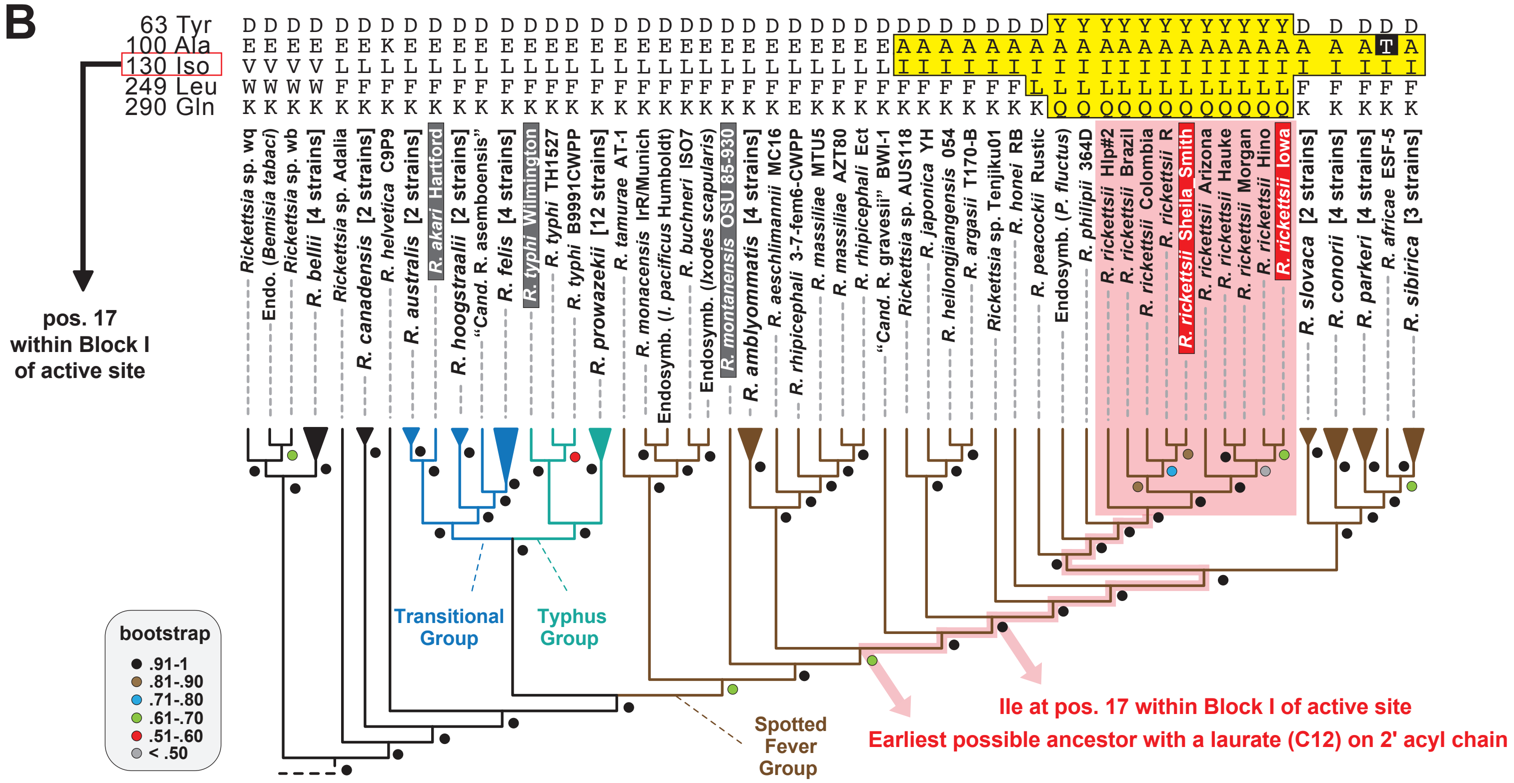

Supplement: FIG S4 [file mSphere.00184-21-sf004.pdf]
