# Supplementary material for: Lipid A Structural Divergence in Rickettsia Pathogens
Source: mSphere. 2021 May 5;6(3):e00184-21. doi: 10.1128/mSphere.00184-21 (PMC8103985; doi:10.1128/mSphere.00184-21)
Supplement: FIG S3 [file mSphere.00184-21-sf003.pdf]

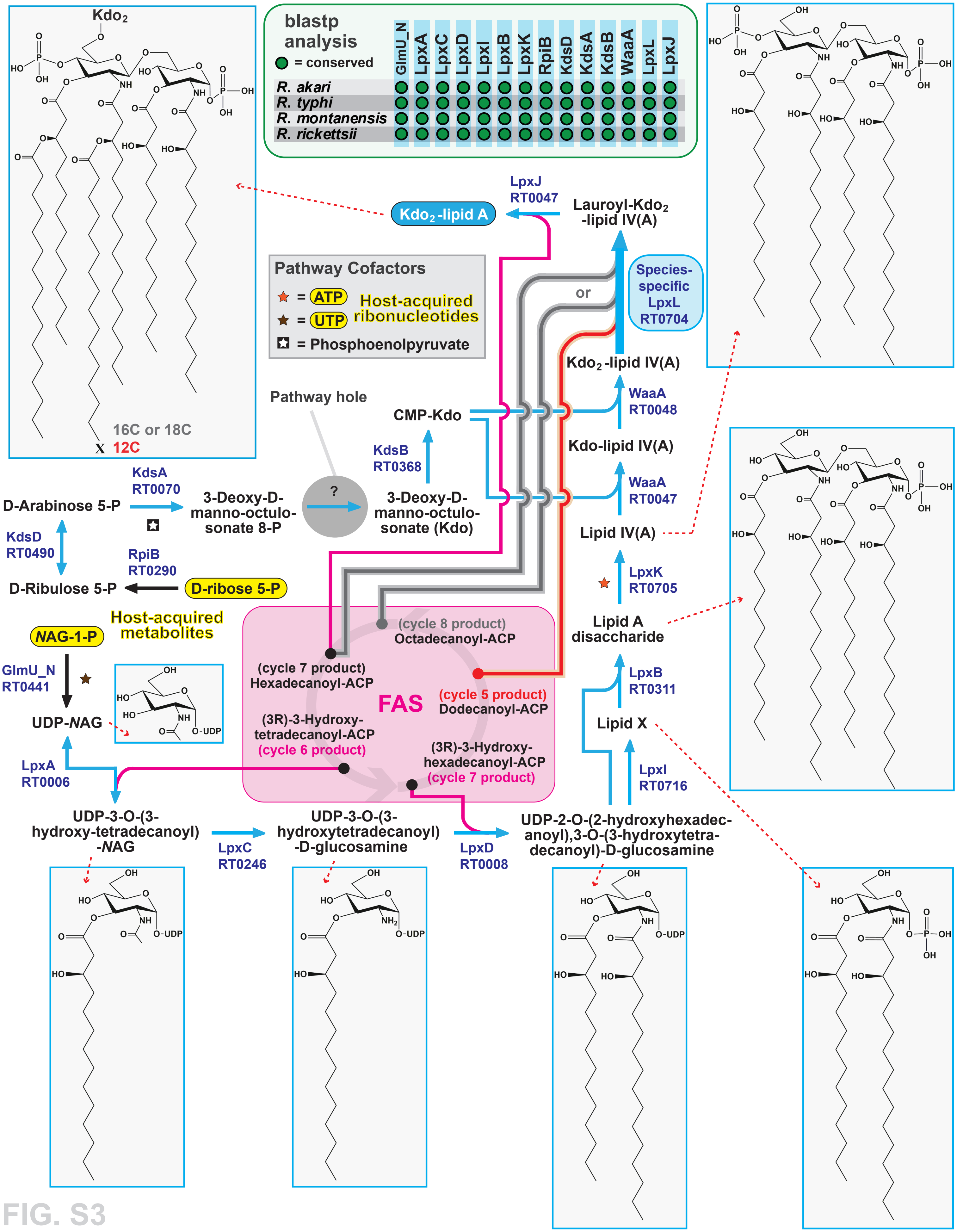

Kdo2
16C or 18C
X 12C
blastp analysis
= conserved
GlmU_N
LpxA
LpxC
LpxD
LpxI
LpxB
LpxK
RpiB
KdsD
KdsA
KdsB
WaaA
LpxL
LpxJ
R. akari
R. typhi
R. montanensis
R. rickettsii
LpxJ
RT0047
Kdo2-lipid A
Lauroyl-Kdo2
-lipid IV(A)
Pathway Cofactors
= ATP
= UTP
Host-acquired ribonucleotides
= Phosphoenolpyruvate
or
Species-specific LpxL RT0704
Kdo2-lipid IV(A)
Pathway hole
CMP-Kdo
WaaA
RT0048
Kdo-lipid IV(A)
KdsB
RT0368
WaaA
RT0047
KdsA
RT0070
D-Arabinose 5-P
3-Deoxy-D-manno-octulo-sonate 8-P
?
3-Deoxy-D-manno-octulo-sonate (Kdo)
Lipid IV(A)
KdsD
RT0490
RpiB
RT0290
D-Ribulose 5-P
D-ribose 5-P
LpxK
RT0705
Host-acquired metabolites
NAG-1-P
(cycle 8 product)
Octadecanoyl-ACP
Lipid A disaccharide
GlmU_N
RT0441
(cycle 7 product)
Hexadecanoyl-ACP
FAS
(cycle 5 product)
Dodecanoyl-ACP
LpxB
RT0311
UDP-NAG
(3R)-3-Hydroxy-tetradecanoyl-ACP
(cycle 6 product)
Lipid X
(3R)-3-Hydroxy-hexadecanoyl-ACP
(cycle 7 product)
LpxI
RT0716
LpxA
RT0006
UDP-3-O-(3-hydroxy-tetradecanoyl)-NAG
LpxC
RT0246
UDP-3-O-(3-hydroxytetradecanoyl)-D-glucosamine
LpxD
RT0008
UDP-2-O-(2-hydroxyhexadec-anoyl),3-O-(3-hydroxytetra-decanoyl)-D-glucosamine

FIG. S3
